# Limited genomic divergence between intraspecific forms of *Culex pipiens* under different ecological pressures

**DOI:** 10.1186/s12862-015-0477-z

**Published:** 2015-09-16

**Authors:** Bruno Gomes, Craig S. Wilding, David Weetman, Carla A. Sousa, Maria T. Novo, Harry M. Savage, António P. G. Almeida, João Pinto, Martin J. Donnelly

**Affiliations:** Global Health & Tropical Medicine, Instituto de Higiene e Medicina Tropical, Universidade Nova de Lisboa, Rua da Junqueira 100, 1349-008 Lisbon, Portugal; Department of Vector Biology, Liverpool School of Tropical Medicine, Pembroke Place, Liverpool, L3 5QA UK; School of Natural Sciences and Psychology, Liverpool John Moores University, Liverpool, L3 3AF UK; Centers for Disease Control and Prevention, 3156 Rampart Road, Fort Collins, CO 80521 USA

## Abstract

**Background:**

Divergent selection can be a major driver of ecological speciation. In insects of medical importance, understanding the speciation process is both of academic interest and public health importance. In the West Nile virus vector *Culex pipiens*, intraspecific pipiens and molestus forms vary in ecological and physiological traits. Populations of each form appear to share recent common ancestry but patterns of genetic differentiation across the genome remain unknown. Here, we undertook an AFLP genome scan on samples collected from both sympatric and allopatric populations from Europe and the USA to quantify the extent of genomic differentiation between the two forms.

**Results:**

The forms were clearly differentiated but each exhibited major population sub-structuring between continents. Divergence between pipiens and molestus forms from USA was higher than in both inter- and intra-continental comparisons with European samples. The proportion of outlier loci between pipiens and molestus (≈3 %) was low but consistent in both continents, and similar to those observed between sibling species of other mosquito species which exhibit contemporary gene flow. Only two of the outlier loci were shared between inter-form comparisons made within Europe and USA.

**Conclusion:**

This study supports the molestus and pipiens status as distinct evolutionary entities with low genomic divergence. The low number of shared divergent loci between continents suggests a relatively limited number of genomic regions determining key typological traits likely to be driving incipient speciation and/or adaptation of molestus to anthropogenic habitats.

**Electronic supplementary material:**

The online version of this article (doi:10.1186/s12862-015-0477-z) contains supplementary material, which is available to authorized users.

## Background

Divergent selection is a major driving force in speciation models involving taxa with overlapping geographic distributions, either during sympatric speciation or via reinforcement of isolation between allopatric incipient species after secondary contact [1, 2]. The capacity for divergent selection to promote reproductive isolation among populations depends on the strength of selection, the number of traits upon which selection is acting and the rates of realised gene flow [3]. Initially, Wu [4] proposed that only strong selection concentrated on a few traits may overcome substantial gene flow, at least for those specific genomic regions which initiate sympatric speciation. However, recent studies have shown much wider divergence across numerous genomic regions between closely related insect ecotypes [5–7].

In insects of medical importance, the speciation process may also have a public health dimension. *Culex pipiens sensu stricto* is a widespread mosquito species with an important medical and veterinary impact owing to its role in the transmission of arthropod-borne viruses (arboviruses) such as the potentially-fatal zoonotic West Nile virus [8]. *Culex pipiens s.s.* is comprised of two distinct forms, denoted pipiens and molestus, which are morphologically indistinguishable but exhibit behavioural and physiological differences that are likely to impact pathogen-transmission. The molestus form is differentiated from pipiens by four key ecological/physiological characteristics: autogeny (the capacity to lay eggs without taking a blood meal), stenogamy (the capacity to mate in confined spaces), homodynamy (a continuous life cycle without diapause), and mammophily (a preference to feed on mammals, including humans) [9, 10].

In southern European/Mediterranean regions, the two *Cx. pipiens s.s.* forms are sympatric in aboveground habitats, but in northern regions of Europe, Russia and the USA, molestus and pipiens segregate into underground and aboveground habitats, respectively [11–13]. A continuous life cycle may be a limitation for surviving in colder climates which may restrain the habitat choice of molestus, while autogeny and stenogamy are important traits for survival in confined underground habitats with restricted access to blood meals. Genomic regions associated with these differentiated traits are currently unknown, as is the degree of ecologically-driven genomic divergence between the forms.

Populations with mixed characteristics between molestus and pipiens have been found in southern European regions [13–15] where inter-form gene flow has been detected, resulting in a pattern of asymmetric introgression from molestus into pipiens [13, 16]. Moreover, an unusual biting preference for birds has been described in the molestus form in southern Europe [17]. Populations with mixed characteristics were also found in USA [18].

Two hypotheses have been proposed for the origin of molestus and pipiens forms. One that the molestus form is polyphyletic; derived from the pipiens form through multiple independent adaptations to underground anthropogenic habitats [11]. The second hypothesis considers molestus as an evolutionarily independent entity from southern latitudes, which has secondarily colonized northern underground habitats [12]. Microsatellite-based studies showed common ancestry of geographically distinct populations of molestus, supporting its status as a single evolutionary entity [12]. However, these studies did not compare aboveground European molestus (in sympatry with pipiens form) and American underground molestus with other geographic populations of this form.

In this study, we performed an AFLP-based genome scan on geographically-distinct *Cx. pipiens s.s.* samples. The main goals of this study were: i) to determine if European and American populations of each form present similar genetic backgrounds; ii) to infer the divergence between molestus and pipiens forms by *F*_*ST*_ estimates; and iii) to quantify outlier rates in inter-form comparisons. Our results provide an insight into how the genetic background of pipiens and molestus forms varies based on their geography and population characteristics (natural/colony populations). This information is crucial for understanding the impacts of habitat adaptation and ecological speciation within this species.

## Results

### Dominant markers and error rates

A total of 894 dominant markers were obtained from 12 primer combinations used in the selective amplification (see Additional file 1: Tables S1 and S2). The markers obtained by the primer combinations EcoRI-ACG/MseI-CGA (Mix1D3) and EcoRI-ACG/MseI-ACC (Mix3D3) yielded high proportions of mismatches between replicates (12.50 and 19.58 %, respectively) and were removed prior to subsequent analysis. The proportion of mismatches from the remaining 810 dominant markers varied between 0.00 and 1.02 % (mean: 0.33 %). Error rates for these 10 primer combinations averaged 1.41 and 0.04 % for the probabilities calculated by AFLPscore [19] of mis-scoring a peak as absent if present, and *vice versa.* Error rates for each primer combination are detailed in Additional file 1: Table S2.

The dataset showed an average of 81 loci per primer-combination with only two combinations yielding more than 100 loci (EcoRI-CTC/MseI-CAA – Mix2D4, EcoRI-CTC/MseI-AGT – Mix4D4; Table S2). The 810 loci presented a balanced distribution among fragment size groups: 172 loci (21.2 %) exhibited small fragment sizes (<125 bp) and 233 loci (28.8 %) largest fragment size (>299 bp), with all remaining fragments 125–299 bp. This dataset complies with the technical recommendation to avoid an imbalanced number of loci per primer-combination and an excessive proportion of loci of small fragment size, thus reducing potential for peak size homoplasy [20].

### Population clustering analysis

**Fig. 1:**
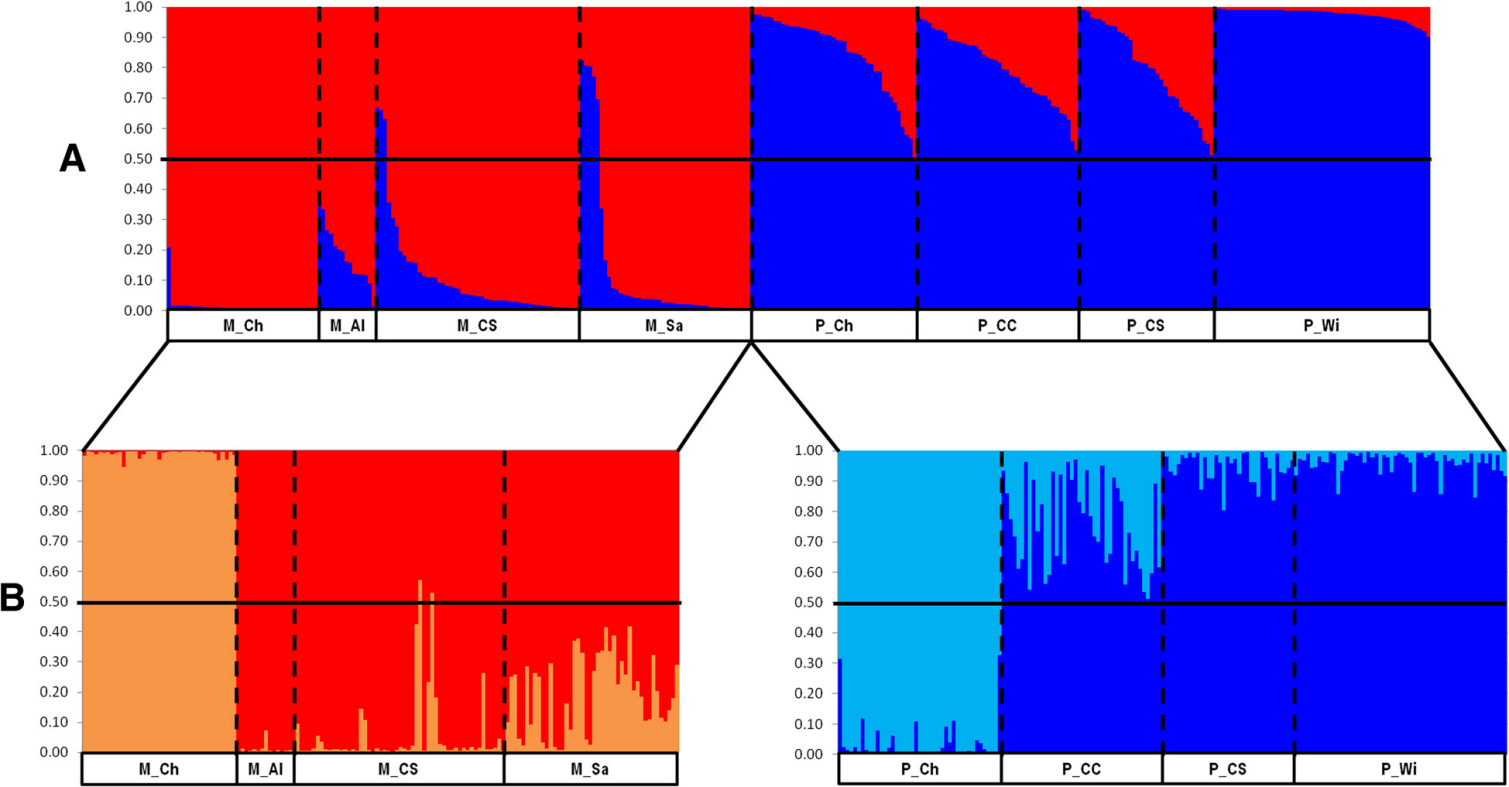
Bayesian cluster analysis conducted by STRUCTURE [21]. **a** analysis with the eight populations of *Cx. pipiens s.s.* **b** analysis within the populations of each form. M_Ch: molestus from Chicago; M_Al: molestus from Alqueva; M_CS: molestus from Comporta, collected inside shelters; M_Sa: molestus from Sandim; P_Ch: pipiens from Chicago; P_CC: pipiens from Comporta, collected in trees by CDC light traps; P_CS: pipiens from Comporta, collected inside shelters; P_Wi: pipiens from Wirral. Columns correspond to the multilocus genotype of each individual, partitioned in different colours representing the probability of ancestry (*q*_*i*_) to each cluster. Individuals were grouped according to their geographic location. Lines indicate the *q*_*i*_ = 0.50

**Fig. 2:**
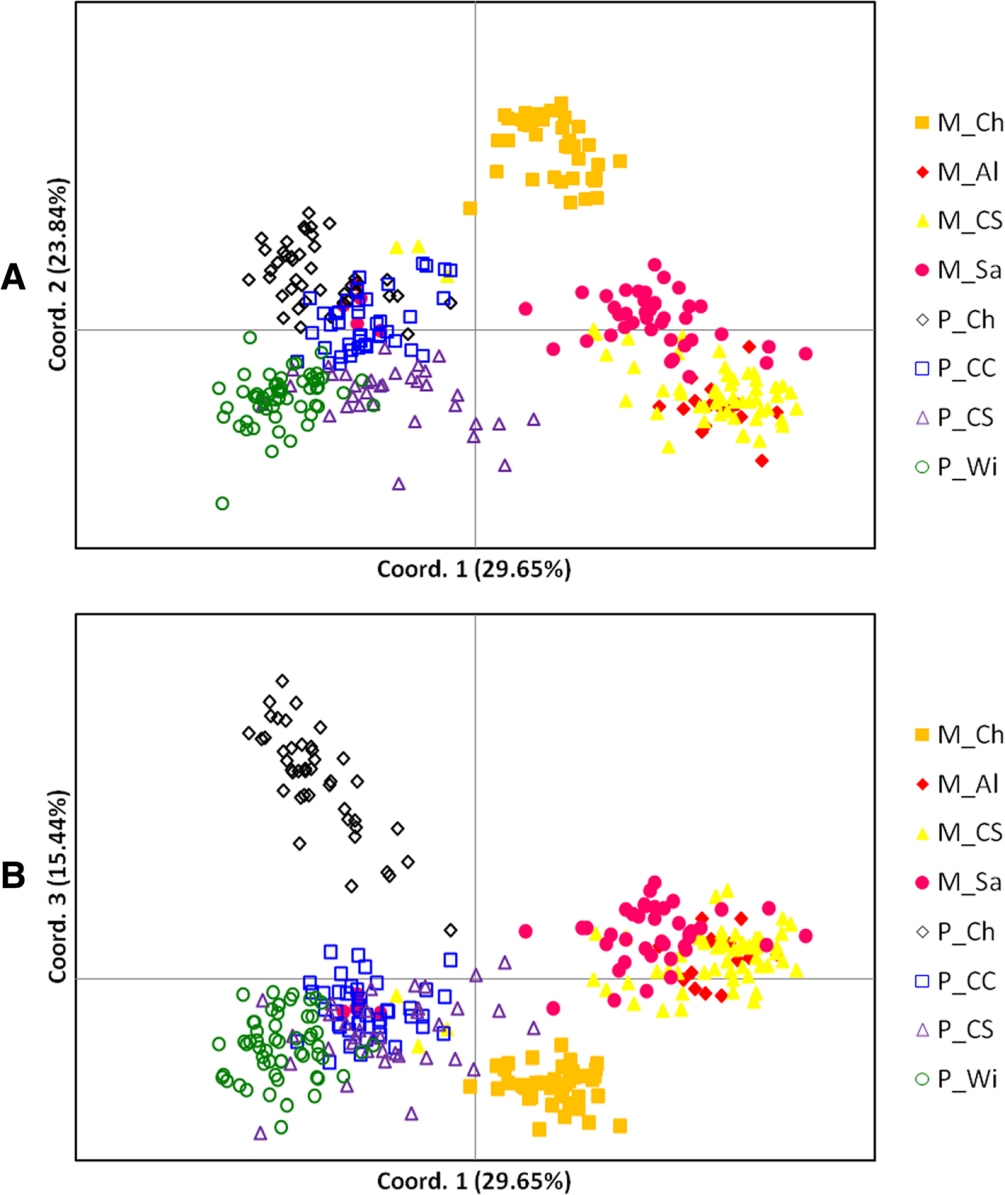
Principal Coordinates Analysis of the eight *Cx. pipiens s.s*. samples conducted by GENALEX 6.41 [22]. **a** two-dimensional plots of principal coordinates 1 and 2; **b** two-dimensional plots of principal coordinates 1 and 3. M_Ch: molestus from Chicago; M_Al: molestus from Alqueva; M_CS: molestus from Comporta, collected inside shelters; M_Sa: molestus from Sandim; P_Ch: pipiens from Chicago; P_CC: pipiens from Comporta, collected in trees by CDC light traps; P_CS: pipiens from Comporta, collected inside shelters; P_Wi: pipiens from Wirral. Coord: coordinate (percentage of variation explained by each coordinate)

STRUCTURE [21] analysis of all 327 female mosquitoes analysed for the 810 loci indicated an optimum of two clusters (see Additional file 1: Fig. S1). Division into the two clusters closely matched the previous form-identification used to select the mosquito samples (full description in Methods, Mosquito samples). However, eight individuals previously identified as molestus (five from Sandim and three from Comporta) presented an individual assignment inferior to 0.50 for this cluster (Fig. 1a). Principal component analysis (PCA) performed by GENALEX 6.41 [22] confirmed the division between the two forms and the placement of the eight previously-identified molestus females closer to pipiens form individuals (Fig. 2). These eight individuals were excluded from the subsequent analysis owing to the conflicting classification between the AFLP data and the other identification methods.

The average probability of membership (*Av.q*_*i*_) obtained by the STRUCTURE varied among geographic samples. In pipiens samples, the UK sample showed a higher *Av.q*_*i*_ (0.976) than the Chicago (USA) sample (0.850) and Comporta (PT) samples (0.779–0.800). In the molestus form, Portuguese samples displayed lower *Av.q*_*i*_ (0.820–0.893) than the Chicago (USA) sample (0.985). The consistently lower *Av.q*_*i*_ in Portuguese samples suggests a higher degree of admixture than in the other geographic samples.

**Fig. 3:**
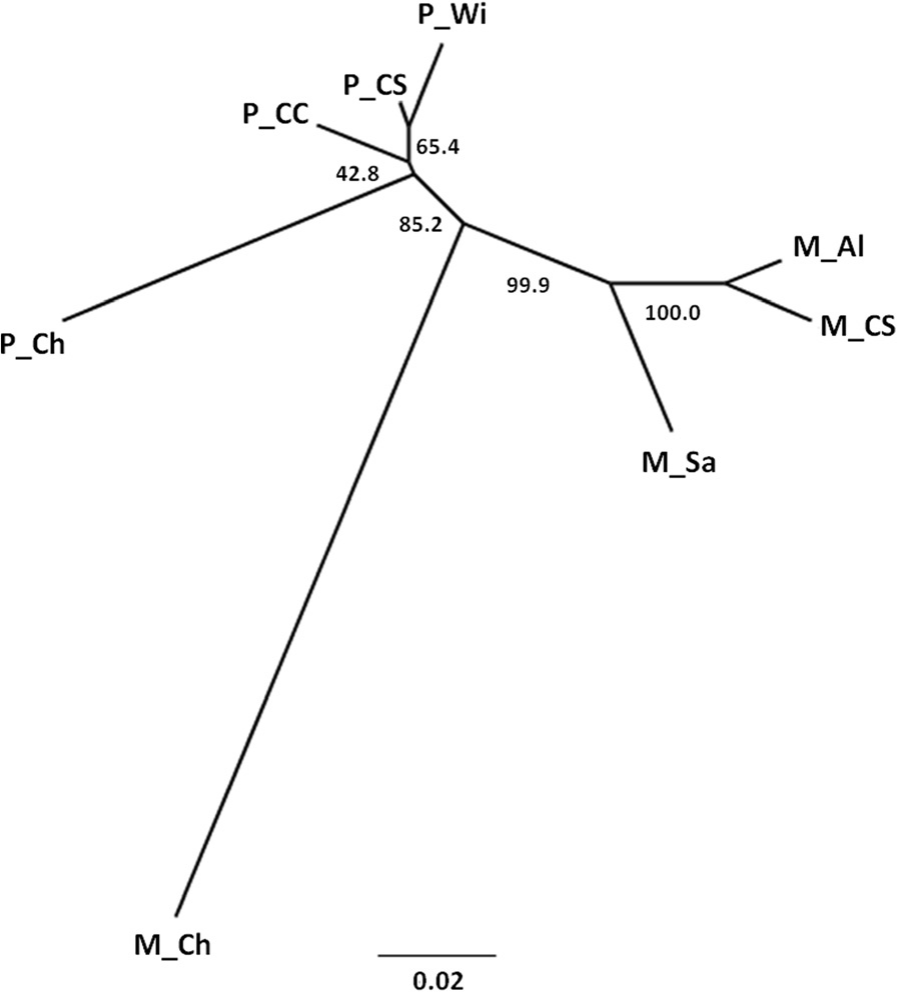
Unrooted Neighbour-joining tree based on *F*_*ST*_ values. Bootstrap (%) support of each branch is given. M_Ch: molestus from Chicago; M_Al: molestus from Alqueva; M_CS: molestus from Comporta, collected inside shelters; M_Sa: molestus from Sandim; P_Ch: pipiens from Chicago; P_CC: pipiens from Comporta, collected in trees by CDC light traps; P_CS: pipiens from Comporta, collected inside shelters; P_Wi: pipiens from Wirral

Clustering analysis was also performed within each form separately; both analyses indicated a division into two clusters, which split Chicago samples from European samples, within each form (Fig. 1b and Additional file 1: Fig. S1). PCA supported the geographic (continental) division within molestus (Fig. 2a) and pipiens (Fig. 2b), with European samples of each form comprising a single group but the samples from Chicago (USA) separated from all the other samples. A neighbour-joining tree based on *F*_*ST*_ values supported the division between the forms and also a high differentiation between the European and American samples, especially in the molestus form (Fig. 3).

AMOVA [23] apportioned 11.7 % of the molecular variance among populations, and only 5.9 % of the variation between the two forms. When the analysis was repeated using European samples alone, the molecular variance among populations fell to 5.6 %, whereas between forms increased to 8.4 %.

Diversity estimates were calculated for each population by the AFLP-SURV [24] (see Additional file 1: Table S3). No significant differences were found between pipiens and molestus forms in number of polymorphic loci (Loc_P: *χ*^*2*^ = 2.09; d.f. = 2; *P =* 0.35), and the proportion of polymorphic loci at the 5 % level (PLP: *χ*^*2*^ = 0.25; d.f. = 2; *P =* 0.88; Additional file 1: Table S3).

### Population differentiation

The *F*_*ST*_ estimates (i.e., mean, median, maximum and three percentiles) used to map divergence among four subsamples presented in our data set (i.e., pipiens and molestus from Europe and USA, respectively) are shown in Table 1 and the Additional file 1: Tables S4 and S5. Comparative pairwise analyses were performed using mean/median and maximum values because those for lower percentiles and the minimum values were negative values of *F*_*ST*_*.*

**Table 1:** Divergence estimates of *F*_*ST*_ pairwise sample analysis per locus

	Molestus			Pipiens			Molestus vs. pipiens					
	All	EU	EU*vs*USA	All	EU	EU*vs*USA	All	EU	USA	EU*vs*USA	EU*vs*M_Ch	EU*vs*P_Ch
Max	0.940	0.597	0.940	0.793	0.793	0.750	0.942	0.806	0.942	0.938	0.938	0.837
Per 99	0.595	0.346	0.718	0.320	0.204	0.355	0.511	0.433	0.798	0.596	0.648	0.553
Per 95	0.261	0.159	0.386	0.137	0.083	0.177	0.241	0.201	0.357	0.288	0.261	0.320
Per 75	0.065	0.038	0.092	0.032	0.015	0.054	0.060	0.054	0.121	0.076	0.069	0.096
Median	0.008	0.004	0.021	0.005	−0.001	0.013	0.014	0.012	0.029	0.022	0.025	0.021
Mean	0.052	0.028	0.080	0.027	0.014	0.041	0.051	0.041	0.091	0.064	0.060	0.069
*N*	3,038	1,621	1,417	4,401	2,296	2,105	10,492	6,658	404	3,711	2,075	1,636

All: within all pairwise comparison; EU: pairwise comparison within European samples; USA: pairwise comparison within USA samples; EU*vs*USA: pairwise comparison between European and USA samples; EU*vs*M_Ch: pairwise comparison between European samples and molestus from Chicago; EU*vs*P_Ch: pairwise comparison between European samples and pipiens from Chicago; Max: maximum *F*_*ST*_ value; Per X: percentile X% of the *F*_*ST*_ values distribution; *N*: total number of pairwise comparison

For almost every measurement of *F*_*ST*_ intra-form comparisons were consistently higher between USA and Europe than among populations within Europe (pipiens: ≈2.3× higher on average; molestus: ≈2.8× higher on average). Between pipiens and molestus forms, *F*_*ST*_ values were higher between the USA samples than in any intercontinental comparison between Europe and USA (≈1.3×). Inter-form comparisons between European samples yielded lower *F*_*ST*_ values than USA (≈1.9×) and intercontinental comparisons (≈1.5×).

In 5 out of 6 comparisons involving the USA molestus sample higher *F*_*ST*_ values were found in intra-form comparisons than in inter-form comparisons with European samples (≈1.2×; Table 1). The high divergence found between intercontinental samples of molestus is illustrated in the neighbour-joining tree (Fig. 3).

### Detecting outlier loci

**Fig. 4:**
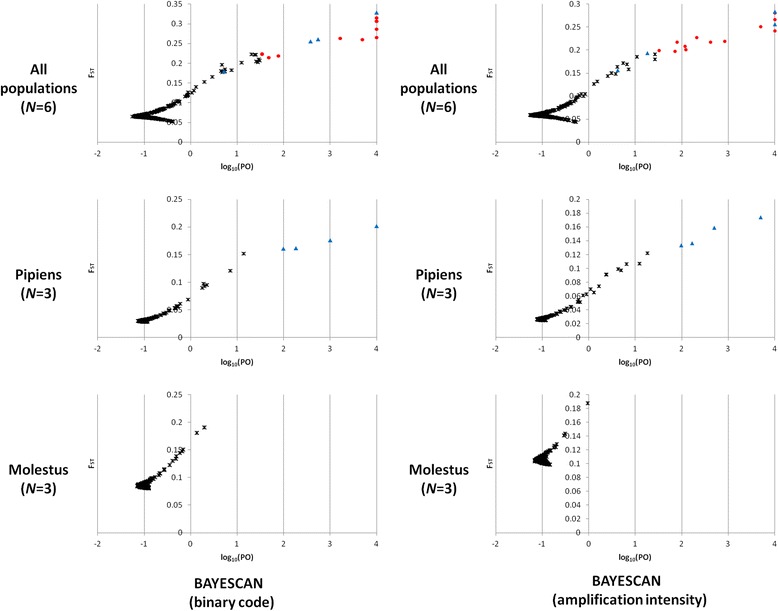
Outlier detection results from BAYESCAN [25, 26] analyses of European populations. *N*: number of samples; Black asterisks: non-outlier loci (log_10_(PO) < 1.5); Blue triangle: outlier loci within form analysis (log_10_(PO) ≥ 1.5); Red dot: outlier loci between pipiens and molestus (log_10_(PO) ≥ 1.5 only for all populations outlier analysis). Note that logarithm of Posterior Odds to base 10 (log_10_(PO)) is arbitrarily fixed to 4 when the posterior probability is 1 (should be infinity)

Due to the marked genetic structure between American and European samples, the detection of outlier loci was performed separately for each continent. Results of the outlier analysis performed using three different approaches among all the European population samples (*N =* 6) and within each form in Europe (*N =* 3) are shown in Fig. 4 and Additional file 1: Fig. S2.

**Fig. 5:**
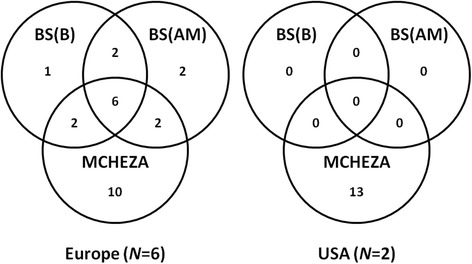
Number of loci detected as outliers in Europe and USA by each method and replicated as outliers in multiple methods. BS(B): BAYESCAN with binary code [25]; BS(AM) BAYESCAN with amplification intensity matrix [26]; MCHEZA: MCHEZA with binary code [27]; *N:* number of samples

In Europe, a total of 25 loci (3.1 %) were scored as outliers across the three methods performed by BAYESCAN 2.1 [25, 26] and MCHEZA [27]. However, this number varied among the methods and only six out of the 25 outlier loci were detected consistently by all methods: Mix1D2_022, Mix3D4_041, Mix4D2_004, Mix4D3_016, Mix4D4_011, and Mix4D4_037 (Fig. 5).

MCHEZA detected 13 (1.6 %) loci as outliers between the molestus and pipiens samples from Chicago (USA) but neither of the approaches implemented by BAYESCAN detected any outliers (Fig. 5). Of the 36 total outlier loci found either in Europe or in USA, only two (0.25 %), Mix3D4_041 (outlier by all methods in Europe) and Mix4D4_027 (outlier only by MCHEZA), were found consistently across both continents (see Additional file 1: Table S6).

However, USA samples exhibited only half the number of scored polymorphic loci (*N =* 406) compared with European samples. In fact, for six of the within-Europe outliers (Mix1D4_006, Mix1D4_063, Mix2D2_039, Mix4D2_023, M4D2_049, Mix4D3_044) no positive band was detected in the USA samples, precluding its inclusion in the USA analysis. This drastic reduction of polymorphic loci in the USA data set led to a higher proportion of small-sized loci (33.7 %) that significantly changed the loci distribution among fragment size groups when compared with the original (*χ*^*2*^ = 45.83, d.f. = 3, *P* < 0.0001; see Additional file 1: Table S7).

## Discussion

In this study, a rigorously quality-controlled AFLP procedure was applied to understand the nature of differentiation within and between pipiens and molestus forms of *Culex pipiens* from two continents. This genome-wide AFLP scan provides additional evidence supporting the hypothesis that molestus and pipiens forms correspond to evolutionarily distinct entities [12]. Independent of geographic origin, molestus samples clustered together and were genetically distinct from pipiens samples highlighting a common ancestry between European and USA molestus populations. This result was consistent in all analyses of population structure conducted. In addition to the molestus/pipiens partitioning, population sub-structuring was found between continents within each form.

Inter-continental differentiation was higher within the molestus than the pipiens form, possibly due to two factors. 1) Colonization of an underground habitat by the USA molestus population studied (which was a sealed habitat blocking contact with other populations [28]). This may have increased genetic drift associated with founder effects/bottlenecks. High differentiation has also been observed between natural populations of molestus in Chicago and New York [29]. 2) Laboratory colonization of the samples analysed and their maintenance for 2 years is very likely to have inflated differentiation compared to that expected among equivalent natural source populations. However, the molestus form presents ecological traits more adaptable to laboratory conditions (i.e., autogeny and stenogamy) when compared with the pipiens form, which theoretically could lead to higher differentiation in pipiens than molestus when the laboratory colonies were created. Therefore, the highest differentiation observed for the molestus sample of Chicago (USA) may have resulted from the additive effect of underground habitat colonization that isolated this population and the subsequent laboratory colony establishment/maintenance (at a colony density of ≈ 2000 adult specimens, varying between 800 and 3200).

Multilocus screening by AFLP is able to identify candidate loci linked to adaptive genetic variation (i.e., outlier loci) that may be associated with mechanisms of selection and species adaptation [30]. The anonymous random information of AFLP does not allow determining the distribution of outlier loci in the genome but this technique is able to identify consistent signals among geographic populations and estimate the proportion of outlier loci in the genome. On average, the percentage of loci scored as outliers in incipient species comparisons varies between 5 and 10 % (range: 0.4–24.5 %; Michel et al. [5]). Thus, outlier rates between pipiens and molestus appear to be relatively low with 3.1 % (25 outliers in 810 loci) of loci outlying in comparisons among European samples and 1.6 % (13 outliers in 810 loci) between USA samples, with the latter actually being 3.2 % if it is considered that only half as many markers were scored in USA samples (*N =* 406).

Such outlier rates are comparable to those obtained using a SNP-chip (3.6 % of outliers) to compare the genomes of the M and S molecular forms of the malaria vector *Anopheles gambiae s.s.* (now named as *Anopheles coluzzii* and *Anopheles gambiae s.s.* [31]), from Guinea-Bissau [32]. Interestingly, the proportion of outlier loci found in Guinea-Bissau, an area of exceptionally high hybridisation, was much lower than from inter-form comparisons in Ghana and Cameroon, where gene flow is much lower [32]. Similarity between the outlier rates found in the present analysis in Europe (*Cx. pipiens s.s.*) and Guinea-Bissau (*An. gambiae s.s.*) might be expected since both included the analysis of sympatric mosquito populations with elevated hybridization [13, 33]. Estimates below the average have also been found in other sympatric populations of closely related insects with gene flow [34, 35].

The low genomic divergence in Europe between pipiens and molestus forms (outlier rates of 3.1 % and an *F*_*ST*_ average of 0.041) and the lower *Av.q*_*i*_ in Portuguese samples that indicate a higher background noise in pipiens than molestus samples are consistent with a pattern of asymmetric introgression from molestus into pipiens [36] previously observed in two distinct geographical areas (*ca.* 2700 km apart) of southern Europe with similar landscapes (Comporta and Thessaloniki [13, 16]). Gomes et al. [13] hypothesized that this pattern could be promoted by differences in mating strategies between the *Cx. pipiens s.s.* forms: stenogamous molestus males (i.e., indoor opportunistic behaviour) and eurygamous pipiens males (i.e., outdoor swarm-based specialist behaviour). In fact, indoor mating has been associated with a breakdown of assortative mating between molecular forms of *An. gambiae* [37].

The similar outlier rates of USA and Europe inter-form analysis contrast with the higher *F*_*ST*_ estimates found in USA inter-form comparisons (≈1.9× higher on average) than European inter-form comparisons (Table 1). This low outlier rates in USA may be explained by form-specific signal lost in the molestus sample of Chicago (USA) due to founder effects and genetic drift in their colony establishment and maintenance, a phenomenon previously observed in *Anopheles spp.* laboratory colonies [38]. This pattern is also consistent with high intra/inter-form differentiation observed in colony and field collected molestus of Chicago [29] suggesting that underground colonization may have played a role in this divergence pattern.

When the two inter-form outlier analyses (European and USA) were compared, only two loci (0.25 %), Mix3D4_41 and Mix4D4_027, were found with a consistent outlier signal in both Europe and USA. These loci are likely to be associated with genomic regions involved in ecological speciation and/or in the adaptation to anthropogenic habitats by the molestus form. The capacity of molestus to occupy underground habitats associated with humans, such as subways, sewers and caves [11], has been promoted by stenogamy and autogeny, which allow a continuous existence in confined habitats with low availability of blood meal sources. These traits are retained even when the molestus form coexists with the pipiens form in aboveground habitats, such as in the case of Comporta, Portugal [13]. Likewise, there was a tendency for molestus individuals to occupy aboveground indoor habitats in this region [39]. In mosquitoes, habitat segregation has been considered a major factor underlying the divergence between the M and S forms of *An. gambiae s.s.* [40, 41]. Ecological postmating barriers are expected to act against maladapted hybrids in the alternate M versus S larval habitats [42]. Moreover, autogeny and overwintering diapause are ecological traits essential to survive under non-ideal conditions (i.e., low host availability and low temperature) that may lead to energetic costs [43, 44]. These two ecological traits may play an important role in ecological postmating barriers acting against maladapted hybrids of *Cx. pipiens s.s.* forms.

## Conclusion

This study supports the status of the molestus and pipiens forms as distinct evolutionary entities with low genomic divergence that are likely to be in a process of incipient speciation. However, the anonymous information (i.e., lack of sequence) given by AFLP screening makes identification of genomic regions, genes, and mutations involved in the adaptation and speciation process difficult [30]. Further studies focusing on additional natural populations of *Cx. pipiens* forms, using higher resolution genomic scans with high-throughput technologies are required in order to fully understand the genomic patterns in *Cx. pipiens s.s.* and identify processes that may be involved in the incipient speciation and habitat adaptation of pipiens and molestus forms.

## Methods

### Mosquito samples

Six field-collected samples from Europe were analysed; five from Portugal and one from the United Kingdom (UK). In addition, two USA samples were obtained from laboratory colonies (Table 2).

**Table 2:** Localities of the samples used in the AFLP protocol

Country	Locality	Latitude	Longitude	Year	Method	Form	Insectary	Code	*N*	Ref
Portugal	Alqueva	38°17′54″N	7°35′17″W	2007	IR	molestus^a^	Au/St	M_Al	15	-
				2010	CDC	pipiens	-	P_CC	42	[39]
	Comporta	38°21′09″N	8°46′51″W	2005–2006	IR	molestus	Au/St	M_CS	50	[13]
				2005–2006		pipiens	N-Au/N-St	P_CS	35	[13]
	Sandim	41°01′19″N	8°30′20″W	2010	IR	molestus^a^	-	M_Sa	39	-
UK	Wirral	53°17′24″N	3°02′01″W	2010	IR-I	pipiens^a^	-	P_Wi	56	-
USA	Chicago	41°43′09″N	87°45′23″W	2010	MA	pipiens	N-Au/N-St	P_Ch	43	-
		41°39′49″N	87°36′30″W	2009	BA/LC	molestus	Au/St	M_Ch	39	[28]

Year: collection year and establishment of laboratory colony in USA. IR: Indoor resting collection with mechanical aspirators; IR-I: Indoor resting collections using insecticide spraying; CDC: outdoor collections performed by CDC light traps in trees; MA: collections using hand-held mechanical aspirators (Clarke, Roselle, IL, USA); BA: Collections by backpack aspirator (Model 1412; BioQuip, Rancho Dominguez, CA, USA); LC: larvae collections using dippers. Form: identification based in a combination of molecular analysis and ecological data; ^a^specimens provisionally identified by the CQ11FL marker. Insectary: insectary experiments performed to determine autogeny and stenogamy [13, 28]; Au: autogenous; N-Au: non-autogenous; St: stenogamous; N-St: non-stenogamous. *N*: sample size. Ref: References

The USA form-specific colonies were established from mosquitoes collected in the area of Chicago, IL: molestus, by sampling a drainage sump using backpack aspirators and larval dipping in January 2009 [28]; and pipiens, from overwintering adults sampled by aspiration from a large culvert in January 2010. Both colonies were maintained by the methodology described in Mutebi and Savage [28]. Colonies were maintained at 27.5 °C and 80–90 % relative humidity with light cycle of 14 h light and 10 h of darkness. Larvae were fed with a finely-ground mixture of 39.4 % TetraMin flakes (Tetra Holdings, Blacksburg, VA), 51.7 % liver powder (MP Biomedicals, Solon, OH), and 8.9 % brain/heart infusion (ICN Biomedicals, Aurora, OH), and adult mosquitoes were offered 10 % sucrose solution and raisins. Colonies were maintained separately in 45.7 cm × 45.7 cm (18 in × 18 in) metal cages (BioQuip, Rancho Dominquez, CA) with approximately 2000 (800–3200) adult specimens by the weekly addition of pupae in cups. The pipiens colony was also offered a bloodmeal once per week composed of defibrinated chicken or goose blood (Colorado Serum Company, Denver, CO) using a Hemotek membrane feeding system (Discovery Workshop, Accrington, England). The mosquitoes used in the present study were taken from the colonies in February 2011.

In Portugal, indoor resting mosquito collections with mechanical aspirators were carried out in Comporta between May 2005 and August 2006 [13], in Alqueva (June 2007) and in Sandim (August 2010) (Table 2). A second mosquito collection in Comporta was performed outdoors, using CDC-light traps that were hung in trees, between July and August 2010 [39].

The indoor and outdoor collections carried out in Comporta were characterised with respect to their molestus and pipiens composition by the established microsatellite-based genetic backgrounds associated with particular bioecological traits, as described in previous publications [13, 39] (Table 2). The remaining samples of Alqueva and Sandim were provisionally identified as molestus by a diagnostic size polymorphism in the 5′ flanking region of the CQ11 microsatellite (CQ11FL) [45]. This marker has proven to be useful to identify the presence of molestus and pipiens forms at the population level, but it is only partially effective in discriminating forms at the individual level [13, 16, 39].

The sampling in the UK took place in March 2010, at the veterinary facility of the University of Liverpool, Leahurst, Wirral. Adults overwintering inside farm buildings (a typical behaviour of the pipiens form) were collected by Pyrethrum Spray Collection and were provisionally classified as pipiens by the CQ11FL marker (Table 2).

For all samples, DNA extraction from individual female mosquitoes was performed using the DNeasy blood and tissue kit (Qiagen, Inc., Manchester, UK).

### AFLP protocol

The DNA concentration of each sample was fluorometrically quantified by the Quant-iT™ PicoGreen® dsDNA reagent and kit (Invitrogen™, Paisley, UK) as recommended by Wilding et al. [46].

For each specimen, 100 ng of genomic DNA was used as template in the AFLP protocol described by Wilding et al. [47], but without a dilution step between the ligation and the pre-selective PCRs. Primers used in the amplification are provided in Additional file 1: Table S1. Selective primers were labelled to allow separation of amplified products on a CEQ^TM^ 8000 capillary sequencer (Beckman Coulter Inc., CA, USA) using the Beckman 600 DNA size standard kit – to quantify fragments between 50 and 700 base pairs. Peaks were only called if they exceeded thresholds of both 3 % of the maximum fluorescence peak height and 500 relative fluorescence units of intensity. A raw matrix of the marker peak data was defined using a bin width of 1 bp. These conditions were selected because they showed the highest proportion of reproducible peaks during optimization.

Special precautions were taken in order to avoid misinterpretation due to peak size homoplasy (i.e., lack of homology of co-migrating fragments) [20] and genotyping errors. Automated scoring and replicated samples were used to increase the objectivity of the genotyping process.

The approach of Whitlock et al. [19] was implemented to determine which peaks from the raw data matrix could be scored reliably. A two-step approach, performed by AFLPscore [19], was used to score the peaks from the raw data matrix, with a first step in which the relative threshold in the fluorescence peak height was set at 20 % in order to select the loci from the raw matrix, and a second at 15 % to score the chosen loci. AFLP analysis was repeated on a sub-set of samples for all the primer combinations (Additional file 1: Table S2) to assess technical error using both mismatch rates and Bayesian AFLPscore error analysis (proportion of mismatches; probability of mis-scoring allele 1 as allele 0, denoted *E1;* and probability of mis-scoring allele 0 as allele 1, denoted *E2*) [19].

The number of loci per primer combination and proportion of loci at four fragment size groups (<125 bp; 125–199 bp; 200–299 bp; >299 bp) were determined in order to infer the effects of peak size homoplasy (i.e., lack of homology of co-migrating fragments) in the data set. This phenomenon is one of the major technical challenges in the AFLP technique and may cause overestimation of allele frequencies or reduction in performance for detection of loci under selection. A balanced data set avoiding a high proportion of loci with low fragment size (<125 bp) and high number of loci per combination (>100 loci) is recommended to minimise homoplasy in AFLP data sets [20].

### Population genetic structure and genetic diversity

Bayesian clustering analysis as implemented by STRUCTURE 2.3.3 [21] was used to infer population substructure/ancestry from the AFLP data set without prior information of sampling groups, under conditions of admixture (α allowed to vary between 0 and 10) with allele frequencies correlated among populations (λ set at the default value of 1). Ten independent runs, with 10^5^ iterations during burn-in followed by 20^5^ replications, were performed for each value of *K* (*K* = 1 to 10 clusters for all samples). Information from the output of each *K* (10 runs) was compiled by the Greedy method implemented in CLUMPP [48]. To infer the most likely number of clusters in the sample, two *ad hoc* approaches were implemented by structure harvester v.0.6.94 [49]: i) an estimation of ln[Pr(X|*K*)] [21], and ii) the *ΔK* statistic [50]. Average values of probability of membership per sample (*Av.q*_*i*_) were determined to infer the degree of admixture in each sample.

Divergence among the sampled populations was assessed by analysis of molecular variance (AMOVA [23]) using GENALEX 6.41 [22].

Principal Coordinates Analysis was used to visualise patterns of genetic differentiation among samples in two-dimensional plots. Calculations were performed in GENALEX 6.41 [22] using the standardised covariance method for the distance matrix conversion.

Pairwise estimates of *F*_*ST*_ between collection sites were calculated in AFLP-SURV [24]. To construct a bootstrapped neighbour-joining tree, 10,000 random replicates of pairwise *F*_*ST*_ tables (based on all loci) were calculated also in AFLP-SURV. These tables were used as input for PHYLIP 3.68 [51], in which the programs NEIGHBOR and CONSENSE were used to produce the bootstrapped tree. Figtree v.1.3.1 [52] was used to visualize the tree.

The number of polymorphic loci, proportion of polymorphic loci at the 5 % level, and expected heterozygosity [53] were estimated assuming Hardy-Weinberg equilibrium in AFLP-SURV. Chi-squared tests on contingency tables - available in VassarStats [54] - were performed to assess differences between pipiens and molestus forms for these genetic diversity estimates. To perform a paired chi-square analysis, diversity estimates were averaged between the pipiens samples from Comporta (CDC light traps) and Wirral and compared to the mean of the molestus samples from Alqueva and Sandim.

### Loci divergence and outlier loci detection

BAYESCAN 2.1 [25] was used to compare neutral models with models including selection and to estimate the posterior odds (PO) in support of selection over neutrality for each locus. BAYESCAN was applied to the binary code (i.e., allele presence/absence) typical for dominant markers. A second approach was implemented using the amplification intensity matrix which can provide additional information from AFLP marker data and yield power similar to that of co-dominant markers [26]. We conducted 20 pilot runs with a length of 5000 iterations each followed by an additional burn-in of 50,000 iterations; preceding tests indicated that this was sufficient to achieve convergence in the Markov chain Monte Carlo. Default values were used for sample size (5000) and thinning interval (10). The prior odds were set as 10 as recommended by the manual for data with a few hundred loci. For the amplification intensity matrix we used 0.10 as threshold for the recessive genotype as a fraction of maximum band intensity. Outliers were identified by the direct estimation of a posterior probability for each locus using a reversible-jump Monte Carlo Markov chain (threshold: log_10_ (PO) > 1.5).

The third approach for outlier detection used the DFDIST algorithm [55], as implemented in the software MCHEZA [27]. The DFDIST method compares empirical *F*_*ST*_ values to a null distribution derived from coalescent simulations and determines the probability that observed *F*_*ST*_ values are as large as, or larger than, the observation under neutrality. Runs were conducted under ‘neutral mean *F*_*ST*_’, which involves computing the initial mean *F*_*ST*_ uninfluenced by outliers, with the following default settings: 50,000 simulations; 0.1 false discovery rate; 0.1 theta; 0.25 beta-a; and 0.25 beta-b. The significance threshold for outlier detection was set at ≥0.95 percentile of simulations.

Detection of outlier loci was conducted differently according to the geographic origin of samples. For European samples, outliers were first identified over all six samples and then within molestus and pipiens samples. Outliers identified among all populations, but not among either of the within-form analyses, were considered as candidate loci under divergent selection between pipiens and molestus. This indirect approach could not be applied to the USA samples because only one sample from each form was analysed. Therefore, outliers were identified from the direct comparison between pipiens and molestus samples. The direct approach between two population samples requires a cautious interpretation because outlier detection methods are known to be less robust with a small number of populations for comparison [25].

Pairwise analyses among all populations were performed by MCHEZA in order to map divergence across the *F*_*ST*_ values distribution (i.e., minimum value, mean, median, maximum value and percentiles).

## Additional file

Additional file 1: Table S1. Primers used in the AFLP protocol. **Table S2** Error rates of the loci obtained by each primer combination in the selective amplification. **Table S3.** Population diversity of the eight populations used in the study. **Table S4.** Divergence estimates based on *FST* pairwise sample analysis per locus within pipiens and molestus samples. **Table S5.** Divergence estimates based in *FST* pairwise sample analysis per locus between pipiens and molestus samples. **Table S6.** Loci detected as outliers in each comparative analysis (Europe and USA). **Table S7.** Proportion of the loci by fragment size in the Overall and USA data. **Fig. S1.** Graphics of *ad hoc* approaches to infer the number of clusters (*K*) in STRUCTURE analysis with all samples. **Fig. S2.** Outlier detection results from MCHEZA analyses. (PDF 451 kb)
