# Supplementary material for: Limited genomic divergence between intraspecific forms of Culex pipiens under different ecological pressures
Source: BMC Evol Biol. 2015 Sep 16;15:197. doi: 10.1186/s12862-015-0477-z (PMC4573496; doi:10.1186/s12862-015-0477-z)
Supplement: Additional file 1: Table S1. — Primers used in the AFLP protocol. Table S2 Error rates of the loci obtained by each primer combination in the selective amplification. Table S3. Population diversity of the eight populations used in the study. Table S4. Divergence estimates based on FST pairwise sample analysis per locus within pipiens and molestus samples. Table S5. Divergence estimates based in FST pairwise sample analysis per locus between pipiens and molestus samples. Table S6. Loci detected as outliers in each comparative analysis (Europe and USA). Table S7. Proportion of the loci by fragment size in the Overall and USA data. Fig. S1. Graphics of ad hoc approaches to infer the number of clusters (K) in STRUCTURE analysis with all samples. Fig. S2. Outlier detection results from MCHEZA analyses. (PDF 451 kb) [file 12862_2015_477_MOESM1_ESM.pdf]

Additional File 1

Table S1. Primers used in the AFLP protocol

Primer	Sequence	Extra Base	Label	Stage
EcoRI	GACTGCGTACCAATTC	A		Pre-Selective PCRs
		C		
		ACG	D3 (HEX)	Selective PCRs
		AGT	D2 (NED)	
		CAG	D2 (NED)	
		CTC	D2 (NED)	
		CTC	D4 (6-fam)	
MseI	GATGAGTCCTGAGTAA	A		Pre-Selective PCRs
		C		
		ACC		Selective PCRs
		AGT		
		ATC		
		CAA		
		CCA		
		CGA		
		CTC		
		AGTC		
		CGAG		

Labels D2-D4 are Beckman-Coulter WellRED dyes, with their Applied Biosystem equivalents shown in parentheses

Table S2. Error rates of the loci obtained by each primer combination in the selective amplification

Group	Fluorescence	Primer EcoRI	Primer MseI	SR	Markers	E1 (%)	E2 (%)	M (%)
Mix1	D2 (NED)	EcoRI-CTC	MseI-CTC	16	69	3.02	0.03	0.72
	D3 (HEX)	EcoRI-ACG	MseI-CGA	16	40	4.33	0.06	12.50
	D4 (6-fam)	EcoRI-CTC	MseI-CGA	16	80	1.30	0.06	0.39
Mix2	D2 (NED)	EcoRI-AGT	MseI-CGAG	14	46	0.46	0.03	0.00
	D3 (HEX)	EcoRI-ACG	MseI-ATC	14	59	0.67	0.03	0.12
	D4 (6-fam)	EcoRI-CTC	MseI-CAA	14	108	0.34	0.02	0.07
Mix3	D2 (NED)	EcoRI-CTC	MseI-CCA	19	100	1.08	0.03	0.26
	D3 (HEX)	EcoRI-ACG	MseI-ACC	19	44	0.67	0.03	19.58
	D4 (6-fam)	EcoRI-CTC	MseI-ATC	19	85	0.95	0.04	0.25
Mix4	D2 (NED)	EcoRI-CAG	MseI-CGA	17	81	1.88	0.03	0.44
	D3 (HEX)	EcoRI-ACG	MseI-AGTC	17	46	4.16	0.08	1.02
	D4 (6-fam)	EcoRI-CTC	MseI-AGT	17	136	0.26	0.01	0.04

SR: samples repeated to infer the error analysis; E1: probability of mis-scoring allele 1 as allele 0; E2: probability of mis-scoring allele 0 as allele 1; M: mismatch. Labels D2-D4 are Beckman-Coulter WellRED dyes, with their Applied Biosystem equivalents shown in parentheses

Table S3. Population diversity of the eight populations used in the study

Population	*N*	Form	#loc	#loc_P	PLP	*He*	S.E.(*He*)
M_Ch	39	M	810	129	15.9	0.053	0.004
M_Al	15	M	810	268	33.1	0.113	0.005
M_CS	49 (50)	M	810	253	31.2	0.093	0.005
M_Sa	39	M	810	179	22.1	0.074	0.005
P_Ch	43	P	810	200	24.7	0.080	0.005
P_CC	42	P	810	249	30.7	0.091	0.004
P_CS	34 (35)	P	810	320	39.5	0.116	0.004
P_Wi	55 (56)	P	810	356	44	0.120	0.004

N: number of individuals without missing data (with missing data); #Loc: number of loci; #Loc_P: number of loci with positive bands (*i.e.* polymorphic loci); PLP: proportion of polymorphic loci at the 5% level; *He*: expected heterozygosity; S.E.(*He*): standard error of *He*; M: molestus form; P: pipiens form; M_Ch: molestus from Chicago; M_Al: molestus from Alqueva; M_CS: molestus from Comporta, collected inside shelters; M_Sa: molestus from Sandim; P_Ch: pipiens from Chicago; P_CC: pipiens from Comporta, collected in trees by CDC light traps; P_CS: pipiens from Comporta, collected inside shelters; P_Wi: pipiens from Wirral.

Table S4. Divergence estimates based on F_{ST} pairwise sample analysis per locus within pipiens and molestus samples

	molestus						pipiens					
	M_Al-M_Ch	*M_Al-M_CS*	*M_Al-M_Sa*	*M_Ch-M_CS*	*M_Ch-M_Sa*	*M_CS-M_Sa*	*P_Ch-P_CC*	*P_Ch-P_CS*	*P_Ch-P_Wi*	*P_CC-P_CS*	*P_CC-P_Wi*	*P_CS-P_Wi*
Max	0.933	0.377	0.597	0.940	0.939	0.591	0.587	0.750	0.556	0.793	0.645	0.434
Per 99	0.730	0.298	0.373	0.703	0.678	0.306	0.336	0.383	0.348	0.206	0.231	0.164
Per 95	0.396	0.116	0.199	0.346	0.379	0.160	0.168	0.202	0.174	0.075	0.093	0.080
Per 75	0.113	0.027	0.049	0.090	0.076	0.037	0.046	0.056	0.054	0.012	0.022	0.012
**Median**	**0.038**	**0.001**	**0.003**	**0.023**	**0.020**	**0.005**	**0.008**	**0.012**	**0.017**	**-0.003**	**0.004**	**-0.002**
**Mean**	**0.093**	**0.018**	**0.038**	**0.074**	**0.072**	**0.031**	**0.038**	**0.043**	**0.042**	**0.012**	**0.019**	**0.010**
N	467	576	488	541	409	557	641	704	760	736	771	789

M_Ch: molestus from Chicago; M_Al: molestus from Alqueva; M_CS: molestus from Comporta, collected inside shelters; M_Sa: molestus from Sandim; P_Ch: pipiens from Chicago; P_CC: pipiens from Comporta, collected in trees by CDC light traps; P_CS: pipiens from Comporta, collected inside shelters; P_Wi: pipiens from Wirral. Max: maximum F_{ST} value; Per X: percentile X% of the F_{ST} values distribution; *N*: total number of pairwise comparison.

Table S5. Divergence estimates based in F_{ST} pairwise sample analysis per locus between pipiens and molestus samples

	molestus *vs.* pipiens															
	M_Al-P_Ch	*M_Al-P_CC*	*M_Al-P_CS*	*M_Al-P_Wi*	*M_Ch-P_Ch*	*M_Ch-P_CC*	*M_Ch-P_CS*	*M_Ch-P_Wi*	*M_CS-P_Ch*	*M_CS-P_CC*	*M_CS-P_CS*	*M_CS-P_Wi*	*M_Sa-P_Ch*	*M_Sa-P_CC*	*M_Sa-P_CS*	*M_Sa-P_Wi*
Max	0.817	0.749	0.632	0.730	0.942	0.912	0.938	0.930	0.837	0.806	0.424	0.579	0.701	0.531	0.575	0.548
Per 99	0.692	0.531	0.364	0.494	0.798	0.578	0.655	0.622	0.536	0.430	0.341	0.451	0.484	0.386	0.399	0.407
Per 95	0.320	0.253	0.192	0.248	0.357	0.239	0.250	0.270	0.310	0.227	0.154	0.212	0.320	0.162	0.189	0.217
Per 75	0.114	0.058	0.041	0.056	0.121	0.057	0.057	0.070	0.079	0.051	0.037	0.068	0.081	0.044	0.055	0.063
**Median**	**0.029**	**0.005**	**0.000**	**0.007**	**0.029**	**0.019**	**0.025**	**0.031**	**0.023**	**0.009**	**0.009**	**0.020**	**0.019**	**0.009**	**0.011**	**0.022**
**Mean**	**0.076**	**0.046**	**0.030**	**0.042**	**0.091**	**0.054**	**0.060**	**0.065**	**0.068**	**0.043**	**0.031**	**0.050**	**0.063**	**0.037**	**0.040**	**0.049**
N	554	663	714	772	404	623	695	757	598	686	723	723	484	637	696	763

M_Ch: molestus from Chicago; M_Al: molestus from Alqueva; M_CS: molestus from Comporta, collected inside shelters; M_Sa: molestus from Sandim; P_Ch: pipiens from Chicago; P_CC: pipiens from Comporta, collected in trees by CDC light traps; P_CS: pipiens from Comporta, collected inside shelters; P_Wi: pipiens from Wirral. Max: maximum F_{ST} value; Per X: percentile X% of the F_{ST} values distribution; *N*: total number of pairwise comparison.

Table S6. Loci detected as outliers in each comparative analysis (Europe and USA).

Loci	Europe	USA
Mix3D4_041	X	X
Mix4D4_027	X	X
Mix1D2_011	X	
Mix1D2_021		X
Mix1D2_022	X	
Mix1D2_024	X	
Mix1D4_006	X	
Mix1D4_007	X	
Mix1D4_009		X
Mix1D4_024		X
Mix1D4_054		X
Mix1D4_063	X	
Mix2D2_039	X	
Mix2D3_001	X	
Mix2D4_012	X	
Mix2D4_026	X	
Mix2D4_042	X	
Mix2D4_059	X	
Mix2D4_062	X	
Mix2D4_076	X	
Mix3D2_006		X
Mix3D4_007		X
Mix3D4_017		X
Mix3D4_026	X	
Mix4D2_002	X	
Mix4D2_004	X	
Mix4D2_023	X	
Mix4D2_025		X
Mix4D2_049	X	
Mix4D3_011		X
Mix4D3_016	X	
Mix4D3_044	X	
Mix4D4_011	X	
Mix4D4_026		X
Mix4D4_037	X	
Mix4D4_063		X

Table S7. Proportion of the loci by fragment size in the Overall and USA data

**Fragment size (bp)**	**All**		**USA**	
	N	***p***	***N***	***p***
<125	172	0.212	137	0.337
125-199	199	0.246	121	0.298
200-299	206	0.254	92	0.227
>299	233	0.288	56	0.138
**TOTAL**	810		406	
	$\chi 2$ =45.83, d.f.=3, $P<0.0001$			

N: number of markers; *p*: proportion of marker per category

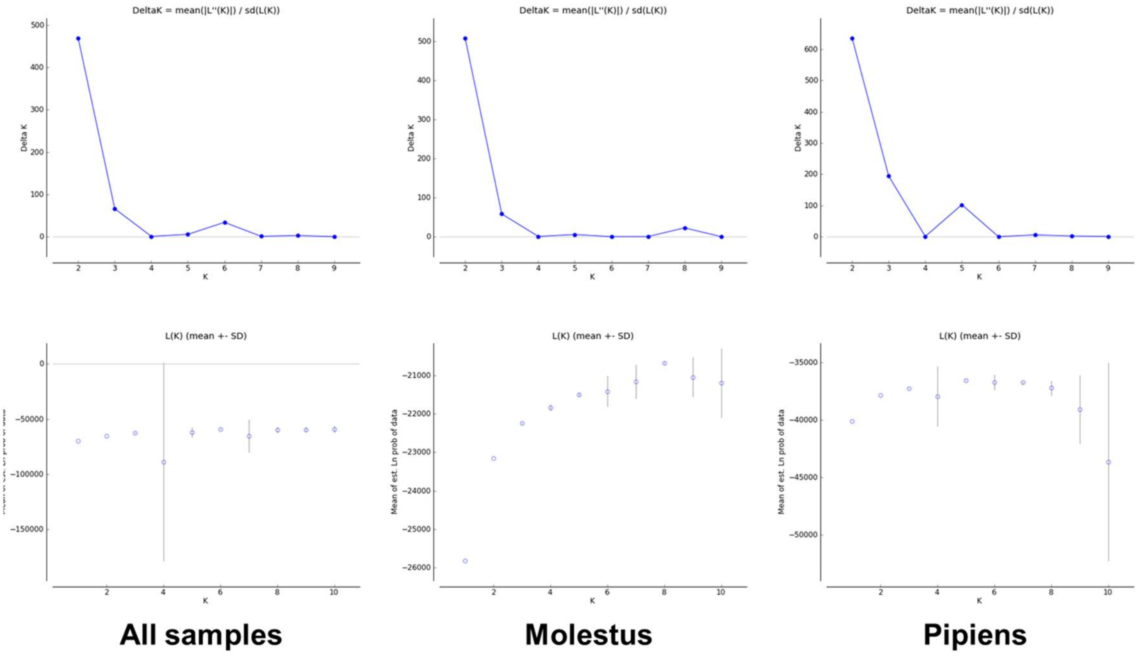

Fig. S1. Graphics of *ad hoc* approaches to infer the number of clusters (*K*) in STRUCTURE analysis with all samples

K: number of clusters; Δ*K*: see Evanno *et al.*, (2005); ln[Pr(X|*K*)]: estimated log probability of the data under each *K*.

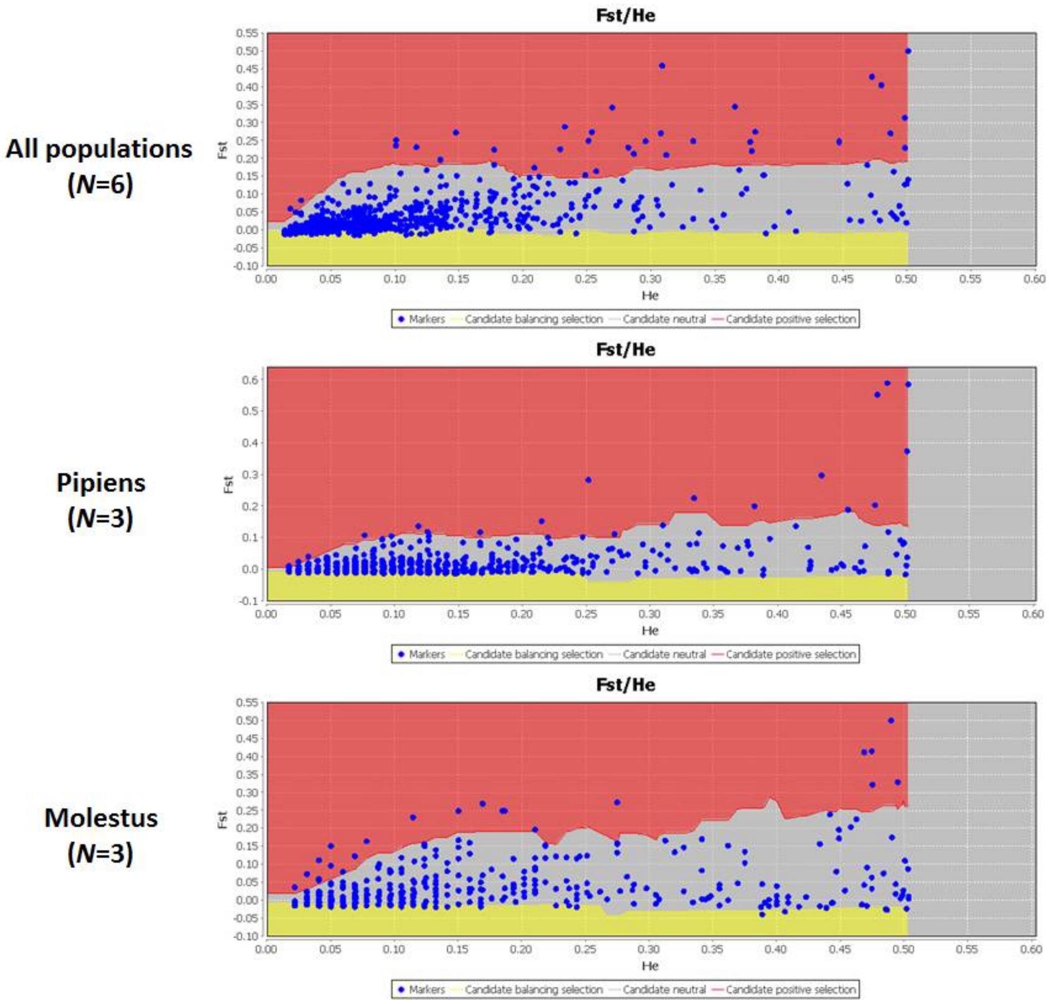

Fig. S2. Outlier detection results from MCHEZA analyses

N: number of samples; Plots show F_{ST} values, conditional on heterozygosity, of the 810 AFLP loci studied. Blue dot: locus; Yellow area: candidate for balancing selection; Red area: Candidate for positive selection (Outliers); Grey area: candidate for neutrality.
